# Self-Induced Mania Methods and Motivations Reported in Online Forums: Observational Qualitative Study

**DOI:** 10.2196/56970

**Published:** 2024-12-06

**Authors:** Emmanuelle CS Bostock, Adriana G Nevarez-Flores, Amanda L Neil, Halley M Pontes, Kenneth C Kirkby

**Affiliations:** 1 Menzies Institute for Medical Research University of Tasmania Hobart Australia; 2 School of Medicine University of Tasmania Hobart Australia; 3 Tasmanian Centre for Mental Health Service Innovation Tasmanian Health Service Hobart Australia; 4 The ALIVE National Centre for Mental Health Research Translation University of Tasmania Hobart Australia; 5 School of Psychological Sciences-Birbeck University of London London United Kingdom

**Keywords:** bipolar disorder, mania, hypomania, self-induced, online forums, consumer reports

## Abstract

**Background:**

In bipolar disorder (BD), mania may be self-induced by manipulation of specific precipitants, as reported in case studies. Another potential source of information on the self-induction of mania is the online postings of users with lived experience of mania.

**Objective:**

The primary aim of this study is to examine the range of methods used to self-induce mania or hypomania described by users of online forums with self-reported BD. Second, we summarize the motivations of users to engage in these behaviors.

**Methods:**

We conducted an observational study of online forum posts that discussed self-induction of mania or hypomania, either in the posters themselves or observed firsthand in others. Posts were identified using Google advanced search operators, then extracted and coded for content in NVivo (version 12 for Mac; QSR International). A total of 44 online forum threads were identified discussing self-induced mania (n=25) or hypomania (n=19). These forums contained 585 posts by 405 usernames, of which 126 usernames discussed methods for self-induction across 327 posts (number of methods per username: median 2, IQR 1-4; range 1-11).

**Results:**

In total, 36 methods were grouped by the authors. The most frequently reported were sleep reduction (n=50), caffeine (n=37), and cessation of medication (n=27). Twenty-six usernames reported their motivation to self-induce mania or hypomania; almost three-quarters (n=19) reported a desire to end a depressive episode. Almost a third of usernames (118/405) explicitly discouraged other forum users from self-inducing mania or hypomania.

**Conclusions:**

Online forums provide an additional and valuable source of information about triggers for mania that may inform relapse prevention in BD. The online forum conversations investigated were generally responsible and included cautionary advice not to pursue these methods.

## Introduction

A self-induced phenomenon refers to one that is “brought about by oneself” [1]. Alternate terms used to describe symptoms, diseases, and neurocognitive and brain states “brought about by oneself” include self-precipitated, self-generated, self-stimulated, and self-regulated. The paradigmatic example of a self-induced phenomenon is self-induced seizures in patients with temporal lobe epilepsy. These seizures are achieved through repeated stimulation including behaviors such as hand waving repeatedly in front of the eyes; light-seeking, or gazing at patterns that may be accompanied by eye fluttering; and head rocking [2]. This stimulation leads to electrical excitement of a seizure focus, accompanied by changes in mood state or consciousness that may be reported as pleasurable. Patients report engaging in these forms of behavior for a variety of motivations, including a pleasant feeling, a reduction in stress or for social advantages [3], and as a treatment for depression [4].

A variety of self-induced symptoms and diseases are reported in psychiatric literature, usually under the rubric of factitious disorders or malingering, including self-induced vomiting [5], water intoxication [6], and neurodermatitis [7]. In factitious disorders, the patient intentionally acts to develop symptoms to assume the sick role, with the motivations unconscious and without external gain [8], whereas in malingering, external gains serve as the primary motivation for the self-induced disorder [8].

Limited attention has been given to self-induced presentations of other psychiatric disorders. One candidate is self-induction of an episode of mania or hypomania in bipolar disorder (BD). BD represents a group of severe and chronic mood disorders that includes bipolar I disorder, defined by the presence of mania, and bipolar II disorder, punctuated by the presence of hypomanic episodes and major depressive episodes [9]. The lifetime prevalence of the bipolar spectrum disorders is estimated at 2.4% of the population [10].

Mania has been defined as “[a] distinct period of abnormally and persistently elevated, expansive, or irritable mood and abnormally and persistently increased activity or energy, lasting at least 1 week” [11]. On the other hand, hypomania lasts at least 4 consecutive days. Contrary to mania, hypomania is less severe and does not cause marked impairment in the individual’s social or occupational functioning and does not necessitate hospitalization [11]. Mania can appear independently, leading to unipolar mania [12,13]. However, it typically appears against the backdrop of preexisting depression that may or may not have received a formal diagnosis [12]. Mania is characterized by relatively rapid onset and offset making it possible to discern proximal precipitating factors for exacerbations or improvements. One consideration is that the external trigger factor may be obscured by prodromal symptoms [14]. An awareness of precipitating factors for mania provides potential avenues for the individual to modify the course of their illness.

In BD, self-induction of the often-pleasurable mood state of mania has only occasionally been reported in psychiatric literature. For example, case reports have described mania self-induced by the use of cough syrup [15] and the abuse of tricyclic antidepressants (dosulepin or clomipramine) [16]. While case reports were the predominant study type in a recent systematic review of triggers of acute mood episodes in BD (86/108 included studies) [17], only 1 study identified the purposeful triggering of manic episodes: a case report pertaining to clinically supervised sleep deprivation [18].

Rodrigues Cordeiro et al [17] identified the most studied trigger of mania in BD as pharmacotherapy, particularly antidepressant use, followed by sleep deprivation, fasting, and stressful life events. Other triggers reported in case studies include neurostimulation, energy drinks, nootropics (eg, acetyl-l-carnitine), herbal medicine (eg, St John’s wort), seasonal changes, the lunar cycle, hormonal changes, and viral infection.

In an earlier narrative review of triggers of mania or hypomania in individuals with BD, Proudfoot et al [19] found a variety of precipitating factors, including goal-attainment events, antidepressant medication changes, disrupted circadian rhythms, spring or summer seasonal conditions, and more tentatively, stressful life events and high emotional expression. Self-induction of mania was not addressed. A detailed appraisal of trigger factors for mania or hypomania and depression in BD was then conducted through a survey of 198 young adults (aged 18-30 years) with BD [20]. Using their custom-made questionnaire “Triggers of mood change in young people with bipolar disorder,” Proudfoot et al [20] reported a total of 21 triggers for mania or hypomania. The 6 most commonly reported triggers of mania or hypomania were falling in love, recreational stimulant use, starting a creative project, late night partying, going on vacation, and listening to loud music. Again, the self-induction of mania was not addressed.

Broad trigger factors for manic episodes were also identified in a study of 207 persons with BD [14]. Of these persons, 44% reported an external trigger, which could be either a positive or negative life event. In order of frequency, the trigger factors identified were work-related factors (eg, starting a new job), medication (including antidepressants), family-related issues, illicit drug use, sleep, and a large grouping of miscellaneous factors. The authors reported that the identification of triggers was not associated with personality traits or the number of affective episodes during a 7-year follow-up period. This study did not examine self-induction of mania or hypomania per se, rather trigger factors in general.

Notably, while both Proudfoot et al [20] and Smedler et al [14] were cited in Rodrigues Corderio et al [17], neither was included as an eligible study, potentially contributing to the noted “lack of large observational studies in the field.” In their reviews of triggers, neither Proudfoot et al [20] nor Smedler et al [14] investigated self-induction as a mechanism linking triggers with the occurrence of mania or hypomania. The self-induction of mania and the methods thereof has not been comprehensively or systematically assessed, necessitating further large observational studies in the field.

People with mental disorders are reportedly eager to form connections with peers through social media, and they report benefits arising from these interactions [21]. Given these interactions, an emerging source of information regarding methods and motivations to self-induce mania or hypomania may be found in self-reported posts in online forums. Online forums are internet sites where users, also referred to as participants or members, may post messages and participate in conversations known as “threads,” which are listed under an opening comment or question referred to as a “thread starter” [22]. Users will then either respond to the initial question posed or to other users’ comments. Online forums can thus provide researchers with rich data sources where users report their own experiences in an open and reflective dialogue, rather than the time-constrained and predominantly closed-question format applied in a treatment setting or questionnaire administration [22].

Online forums have been analyzed in relation to various aspects of BD, including the effect of ketogenic diets on mood [23], online social support and unsolicited advice [24], and self-help [25].

## Methods

### Overview

An observational thematic analysis of posts published in public online forums that related to self-induction of mania or hypomania in BD was conducted. Posts were identified through an advanced Google search undertaken in the third week of September 2021. The search strategy comprised the following syntax: (intitle:induce OR intitle:induced OR intitle:inducing OR intitle:self-induce OR intitle:self-induced OR intitle:trigger OR intitle:“make yourself” OR intitle:deprive) AND (intitle:mania OR inurl:mania OR intitle:“yourself manic” OR intitle:hypomania OR intitle:hypo) AND (inurl:comments OR inurl:threads OR +“Related Questions” OR intitle:Forums OR intitle:diary). Posts that met the inclusion criteria were extracted and threads pertaining to the self-induction of mania or hypomania were downloaded with original formatting retained, and imported into NVivo (version 12; QSR International). As a deidentification procedure, each forum was allocated a study-specific unique ID code, as were posts and participants. Furthermore, no verbatim text from the posts is reported in this publication. The inclusion and exclusion criteria are presented in Textbox 1.

The text content was rated independently by 2 authors (ECSB and AGN-F), who extracted and categorized methods of and motivations for self-induction, as well as whether users were discouraging of the practice. Any disagreements were moderated by a third author (ALN). An initial list of potential triggers was identified based on Proudfoot et al [20] and expanded upon during the data extraction process. Clinical and experiential knowledge was used to classify triggers into nodes using NVivo.

**Inclusion and exclusion criteria for individual posts.**

**Inclusion criteria**

- Posted on a public online forum with a thread dedicated to the subject of self-induction of mania or hypomania
- Written in English
- Accessible without registration or password requirements
- Use of forum data for research purposes was not restricted by the forum's terms and conditions

**Exclusion criteria**

- Defunct URLs
- Posts containing identifying information, such as email addresses
- Posts made by the username “anonymous”
- Deleted posts

### Ethical Considerations

This study conformed to the Australian National Health and Medical Research Council’s National Statement on Ethical Conduct in Human Research (2018) guidelines. This study was granted ethics approval (H0024444) by the University of Tasmania Social Science Ethics Committee. A waiver of consent was sought as there was no way of confirming the identity of a user or of contacting them on the basis of the data recorded within the forums. Forum users were only identified by the usernames they submitted in their posts to that forum and were not linked to communication avenues such as email. Users may or may not have been still posting on a given forum when the posts were identified.

## Results

The search criteria adopted identified 44 online forums with threads on self-induced mania (n=25) or hypomania (n=19). The 44 online forums contained a total of 585 posts with the number of posts per forum ranging from 1-80 (median 10.5, IQR 5.75-17). The 585 posts were attributed to 405 unique usernames (median 1, IQR 1-1; range 1-12). Of these, 126 usernames discussed 1 or more methods to self-induce mania or hypomania (number of methods per username: median 2, IQR 1-4; range 1-11).

The narratives of these 126 usernames contained 327 descriptors related to self-induction, covering a total of 36 methods as grouped by the authors (Table 1). The most widely and frequently reported individual method of self-induction was sleep reduction (forums: n=26; posts: n=50) followed by caffeine (forums: n=22; posts: n=37), then cessation of medication use (forums: n=20; posts: n=27), and antidepressant medication (forums: n=17; posts: n=27). The top 10 methods for self-inducing mania or hypomania accounted for 71.2% (230/323 posts) of all reports of methods of self-induced mania or hypomania across the usernames’ posts. Seven methods (19.4%; N=36) were mentioned only once in the overall sample of posts (ie, nicotine, artificial sweeteners, green tea, playing video games, reduction or cessation of exercise, sexual encounters, and yoga).

**Table 1:** Methods of reported strategies to self-induce mania or hypomania in online forums.

Trigger	Forums, n	Posts, n
Sleep reduction or deprivation	26	50
Caffeine	22	37
Cessation of medication	20	27
Antidepressant medication	17	27
Alcohol	15	19
Prescription stimulants	14	20
Self-imposed psychological demands	14	13
Recreational stimulant use	10	17
Other medication	9	10
Listening to loud music	9	9
Emotion	8	10
Drugs (unspecified)	6	8
Increased exercise	6	7
Diet change	6	6
Energy drinks	6	6
Amino acids	6	5
Cannabis	5	7
Light exposure	5	4
Psychedelics	4	4
Meditation	4	4
Nootropics	4	4
Supplements	4	4
Herbal medicine	3	4
Routine change	3	3
Sugar	3	3
Flu medication	2	2
Isolation	2	2
Psychotropic medications (unspecified)	2	2
Neurostimulation	2	2
Nicotine	1	1
Artificial sweetener	1	1
Green tea	1	1
Playing videogames	1	1
Reduction or cessation of exercise	1	1
Sexual encounters	1	1
Yoga	1	1

Motivations to self-induce mania or hypomania were provided by 26 users and included to relieve a depressive episode (n=19), to validate the diagnosis of BD by inducing mania (n=2) and others (n=5) such as to increase socializing or confidence or to escape reality.

Users typically commented on particular subjects under discussion in a given thread rather than addressing triggers as a whole. In total, 118 of 405 users (29.1%) posted comments explicitly discouraging the practice of self-induction of mania or hypomania.

## Discussion

### Principal Findings

This study is the first to examine the methods and motivations of reported strategies to self-induce mania or hypomania in self-reported BD as posted in online forums, and is the largest sample size reported in a study on self-induced triggers of mania or hypomania in BD. In addition, as one of a growing number of analyses using online (internet) forums as a data source for academic research, this work aids in establishing the usefulness of online forums as a data source, including for lived-experience knowledge and expertise.

This study identified 36 methods for self-inducing mania or hypomania, the largest number of potential triggers of mania or hypomania reported thus far for BD. The top 10 methods for self-inducing mania or hypomania reported by online forum participants, in decreasing order, were sleep reduction, caffeine, cessation of medication, antidepressant medications, alcohol, prescription stimulants, self-imposed psychological demands, recreational stimulants, taking other medications, and listening to music. These methods accounted for 71.2% of all reports of methods of self-induced mania or hypomania across the usernames’ posts.

In comparison with 3 existing studies on triggers of mania in BD (Table 2), the only trigger identified in common across all 4 was antidepressant use. Just over a third of the methods identified within this study (13/36) were explicitly identified as triggers by Proudfoot et al [20], and 2 (including 1 additional method) were explicitly identified by Smedler et al [14]. A further 13 methods identified were potentially captured by the broad classifications of triggers used in the earlier studies, such as “medication” in Smedler et al [14]. Around 15 methods were in common between this study and Rodrigues Cordeiro et al [17], including light exposure and neurostimulation, which had not been identified in either Proudfoot et al [20] or Smedler et al [14]. Newly identified in this study were several lifestyle methods, underpinning the importance of online forums as a standalone source of lived-experience knowledge. These methods were meditation, isolation, playing video games, sexual encounters, and yoga. Notably, increased exercise, meditation, isolation, light exposure, and vagus nerve stimulation were identified in multiple forums, and in turn, in multiple posts.

Differences in methods identified in this study and triggers identified in the earlier literature may reflect the different objectives of the data sources or processes of engagement. For example, forum users report that interacting online can offer personalized care through peers that cannot be provided by health care professionals [26]. Forum users may also experience the “online disinhibition effect,” a phenomenon whereby individuals say and do things online that they would not typically say and do in face-to-face interactions [27], facilitated by open-ended discussion and time for reflection. In contrast, surveys are undertaken by researchers to collect data that may or may not have direct benefits to participants and are subject to time or response constraints. For example, the Proudfoot et al survey [20] mostly listed broad classifications of triggers and a single open-ended question, “We would be delighted if you could come up with any other fascinating or distinct factors which have triggered a high in you. Please tell us about them and also how often they occur.” This question did not lead to the identification of any additional triggers. There may also be a difference between when a person sets out to intentionally induce mania or hypomania and when these conditions are incidentally induced, as in Proudfoot et al [20] and Smedler et al [14]. Online forums also minimize the Hawthorne effect (ie, where participants respond in accordance with perceived expectations of researchers, as they are aware that they are being studied, leading to biased results) [28].

We found sleep deprivation was a trigger for mania or hypomania, consistent with Smedler et al [14] and Rodrigues Cordeiro et al [17]. In contrast, Proudfoot et al [20] found that it was a trigger only for the onset of depression. The study by Proudfoot et al [20] was restricted to young adults (aged 18-30 years), whereas the age range of the online forum sample is not known but may be presumed to include at least older participants (all were required to be adults to participate on forums). Smedler et al [14] conducted a prospective cohort study over 7 years, with a minimum age of 17 years and a mean age of 38 years. Participants with a longer history of BD, and particularly those who have had repeated episodes of mania or hypomania, are better placed to identify methods or trigger factors. The preponderance of depressive symptoms in the average course of BD may also lead to exploration and experimentation of ways to escape depression; hence, the identification of more triggers of mania or hypomania [29].

The wide range of triggers for mania or hypomania reported in the online forums suggests a variety of proximate pathogenetic mechanisms. More than 3 decades ago, Wehr et al [18] hypothesized that diverse psychological, interpersonal, environmental, and pharmacological triggers of mania share a final common pathway of sleep deprivation. Malfunction in the circadian rhythm system has been proposed in the pathogenesis of mania [30,31]. This is supported by our finding that the most reported trigger related to sleep reduction.

While peer-to-peer interactions may involve risks, such as obtaining unreliable information resulting in unrealistic expectations, becoming online dependent, and withdrawing from social activities [21], we found that 118 users explicitly discouraged the practice of self-induction of mania or hypomania. This finding supports the positive aspects of online peer support [26]. However, there are no data available to substantiate whether exposure to discussion about triggers presented in a responsible manner results in improved insight and appropriate behavioral choices. In future research, we recommend the systematic assessment of engagement objectives and practices and an examination of the reasons to seek advice.

**Table 2:** Overlap of methods for self-inducing mania or hypomania reported in online forum posts and triggers for mania or hypomania identified in Proudfoot et al [20], Smedler et al [14] and Rodrigues Cordeiro et al [17].

Methods for self-induction identified in online forums	Proudfoot et al [20]	Smedler et al [14]	Rodrigues Cordeiro et al [17]
Sleep reduction or deprivation	No	Yes	Yes
Caffeine	Yes	No	No
Cessation of medication	No	Broad^a^	Yes
Antidepressant medication	Yes	Yes	Yes
Alcohol	Yes	No	No
Prescription stimulants	No	Broad	Yes
Self-imposed psychological demands	No	Broad	Broad
Recreational stimulant use	Yes	Broad	No
Other medication	No	Broad	Yes
Listening to loud music	Yes	No	No
Emotion	Yes	No	Yes
Drugs (unspecified)	No	Broad	No
Increased exercise	No	No	Yes
Diet change	Yes	No	Yes
Energy drinks	Yes	No	Yes
Amino acids	Broad	No	Yes
Cannabis	Yes	Broad	No
Light exposure	No	No	Yes
Psychedelics	Broad	Broad	No
Meditation	No	No	No
Nootropics	Broad	Broad	Yes
Supplements	Broad	Broad	No
Herbal medicine	Broad	Broad	Yes
Routine change	Yes	No	Yes
Sugar	Yes	No	No
Flu medication	Yes	No	No
Isolation	No	No	No
Psychotropic medications (unspecified)	Broad	Broad	No
Neurostimulation	No	No	Yes
Nicotine	Yes	No	No
Artificial sweeteners	Broad	No	No
Green tea	Broad	No	No
Playing video games	No	No	No
Reduction or cessation of exercise	No	No	No
Sexual encounters	No	No	No
Yoga	No	No	No

^a^“Broad” refers to broad categorizations such as “work” or “miscellaneous.”

### Limitations

This study is limited by several factors. A recognized issue with analyzing online forum accounts of medical conditions is that descriptions are given by the users themselves, without a confirmation of the diagnosis from a health practitioner [25]. Users may not have a formal diagnosis of BD or unipolar mania, and it is accordingly not known for certain how many of the users had a diagnosed medical condition, including BD. Furthermore, it may be the case that some users may have created additional usernames to remain anonymous in the forums they engaged with. The potential bias in people who access online forums constrains the generalizability of the study, and the frequency of these triggers in the general population cannot be assessed.

### Conclusions

This study confirmed the disposition of people with self-reported BD to discuss methods of self-induction of mania or hypomania in online forums, and confirmed that these forums can provide large and wide-ranging sources of data that can lead to novel findings. This study also highlights the importance of the consumer voice in BD research, given that it is, to the best of the authors’ knowledge, the first published study on methods of self-induced mania in BD. An understanding of behaviors that lead to self-induced mania may assist in improving the management and treatment of people with BD and assist in targeting relapse prevention approaches in BD.

## Abbreviations

BD: bipolar disorder

## Data Availability

The data sets generated during or analyzed during this study are available from the corresponding author on reasonable request.
